# ID 137 - Aprimoramento da Avaliação de Tecnologias em Saúde: impactos da atuação da Subcomissão Técnica de Produtos e Procedimentos

**DOI:** 10.5327/2237-9622.2025.v34s1.137

**Published:** 2025-11-25

**Authors:** Aíla Coelho do Carmo, Daniele de Almeida Cardoso, Joana Ferreira da Silva

**Keywords:** Avaliação de Tecnologias em Saúde, ATS, Conitec, Política Nacional de Gestão de Tecnologias em Saúde, Sistema Único de Saúde, SUS, Subcomissão Técnica de Produtos e Procedimentos, Comitê de Produtos e Procedimentos

## Abstract

**Introdução:**

A Subcomissão Técnica de Avaliação de Produtos e Procedimentos estabeleceu-se com a normatização do processo administrativo de incorporação de tecnologias em saúde pela Comissão Nacional de Incorporação de Tecnologias no Sistema Único de Saúde (Conitec), por meio da Portaria GM/MS n.º 4.228, de 6 de dezembro de 2022, a qual altera a Portaria n.º 1, de 28 de setembro de 2017. A abordagem prévia dos assuntos pertinentes a produtos e procedimentos que são discutidos pelo Comitê de Produtos e Procedimentos visa mitigar dúvidas e ressalvas referentes às tecnologias em saúde que poderão impactar no seu processo de recomendação/deliberação, favorecendo a celeridade do trâmite. As avaliações pela Subcomissão ocorrem no fluxo de submissão de incorporação, em especial: na Análise de Conformidade das demandas, na Elaboração do Relatório técnico preliminar e nos retornos após consulta pública para alterações adicionais. Segundo o Painel de acompanhamento do site atual da Conitec, "Conitec em números", no universo das demandas submetidas, 25,9% são tecnologias em saúde tipificadas como produtos e procedimentos entre 2012 e 2024.

O objetivo deste estudo será demonstrar a contribuição das atividades da Subcomissão de Produtos e Procedimentos no aprimoramento das avaliações de demandas de referência submetidas à Conitec a partir de maio de 2023, quando as reuniões da referida subcomissão começaram a ocorrer.

**Método:**

Foi realizada uma pesquisa documental no site atual da Conitec para extrair dados das demandas submetidas à Comissão. Os dados de interesse foram: "Tipo de Tecnologia", "Tema da Saúde ", "Nome da Tecnologia", "Indicação" e "Status". Foram apresentados os dados por meio de frequências e discutidas as principais contribuições das atividades da Subcomissão de Produtos e Procedimentos a partir de maio de 2023.

**Resultados:**

Foram avaliados 3 produtos e 14 procedimentos com o início das atividades da Subcomissão de Produtos e Procedimentos. Os temas em saúde analisados nestas demandas demonstram a amplitude de abrangência, a saber: obstetrícia (5,9%), cardiologia (5,9%), neurologia (17,6%), endocrinologia (5,9%), cardiovascular (5,9%), reumatologia (5,9%), oncologia (35%), hematologia (5,9%), gastroenterologia (5,9%) e infectologia (5,9%). O destaque para as tecnologias oncológicas, com maior frequência, é demonstrado na avaliação da demanda de tomografia computadorizada por emissão de pósitrons (PET-CT) para o tratamento de pacientes com câncer de pulmão de células pequenas, na qual as atividades da Subcomissão contribuíram, de forma ímpar, no levantamento da capacidade instalada.

**Conclusão:**

As demandas de produtos e procedimentos representam uma parcela significativa das avaliações da Conitec. Nesse contexto, o estabelecimento e a condução das atividades pela Subcomissão de Produtos e Procedimentos implicou nas melhorias concretas ao processo de Avaliação de Tecnologias em Saúde (ATS) e na tomada de decisão para os temas de referência, uma vez que: promove articulação interna e externa; antecipa questões de implementação; resolve lacunas de informação sobre a população elegível à demanda; verifica a capacidade assistida e de infraestrutura do SUS; amplia a celeridade nos relatórios e tramitações; e, aprimora dados econômicos e análises de custos.

